# Toward an Ethically Founded Framework for the Use of Mobile Phone Call Detail Records in Health Research

**DOI:** 10.2196/11969

**Published:** 2019-03-22

**Authors:** Kerina Helen Jones, Helen Daniels, Sharon Heys, David Vincent Ford

**Affiliations:** 1 Population Data Science Swansea University Medical School Swansea University Swansea United Kingdom

**Keywords:** mobile phone data, ethical framework

## Abstract

Data derived from the plethora of networked digital devices hold great potential for public benefit. Among these, mobile phone call detail records (CDRs) present novel opportunities for research and are being used in a variety of health geography studies. Research suggests that the public is amenable to the use of anonymized CDRs for research; however, further work is needed to show that such data can be used appropriately. This study works toward an ethically founded data governance framework with social acceptability. Using a multifaceted approach, this study draws upon data governance arrangements in published health research using CDRs, with a consideration of public views and the public’s information expectations from mobile network operators, and data use scenarios of CDRs in health research. The findings were considered against a backdrop of legislative and regulatory requirements. CDRs can be used at various levels of data and geographic granularity and may be integrated with additional, publicly available or restricted datasets. As such, there may be a significant risk of identity disclosure, which must be mitigated with proportionate control measures. An indicative relative risk of the disclosure model is proposed to aid this process. Subsequently, a set of recommendations is presented, including the need for greater transparency, accountability, and incorporation of public views for social acceptability. This study addresses the need for greater clarity and consistency in data governance for CDRs in health research. While recognizing the need to protect commercial interests, we propose that these recommendations be used to contribute toward an ethically founded practical framework to promote the safe, socially acceptable use of CDR data for public benefit. This pattern needs to be repeated for the appropriate use of new and emerging data types from other networking devices and the wider internet of things.

## Introduction

### Background

The number of mobile connections, including the internet of things (IoT), already exceeds the world population and is rapidly approaching 9 billion [1]. It is difficult to find an adequate adjective to represent the magnitude of data being generated as we go about our daily lives. This number will only increase with further technological developments and major investments. Apart from smartphones, advances in smart homes, smart cities, and autonomous vehicles; immersive technologies such as virtual and augmented reality; and wider applications of artificial intelligence are all increasing in permeation [2]. Data derived from our use of the plethora of increasingly connected digital devices hold great potential for public benefit as well as for generating massive income and contributing to economic growth. There is much debate over the definition of public benefit. We define it here as work having real-world value or practical application, with the clear potential to improve the life of individuals or wider society [3]. With such rapidly developing technologies and their societal impact, it is imperative that principles are in place for proper data governance, and such principles are lagging behind the advancing pace [2]. In a seminal paper, Letouzé and colleagues observed the need for mobile phone data, when they noted the absence of a clear holistic ethical and regulatory framework to guide research using call detail records (CDRs) [4].

CDRs are generated passively each time a mobile phone user connects to a mobile network, either by voice call or text message. CDRs are collected irrespective of whether the user has a standard mobile phone or a smartphone that functions as a small personal computer with internet access, social media connectivity, apps, games, etc. The record includes the starting time of the call (or message), its duration, the caller’s and receiver’s phone numbers, and their locations. Locations are estimated from the positions of activated cell towers, but more precise locations can be generated through tower triangulation and Wifi connections. Mobile network operators (MNOs) receive billions of CDRs globally and use them for billing, monitoring data usage, and understanding and targeting customers according to their mobile phone use [5].

Mobile phone CDRs present novel opportunities for research and are being used in a variety of health geography studies. In a recently published article, we reviewed the wide uses of CDRs and showed their benefits in research across many countries, particularly low- and middle-income countries where mobile phones are a lifeline due to the lack of landline connections and population administrative systems [5]. Examples include cholera surveillance in Haiti and the Ivory Coast [6,7], air pollution in Italy [8], visitor movement to monitor malaria in Zanzibar [9], and dengue epidemics in Pakistan [10]. We also conducted research with the public to gain their views on the use of mobile phone CDRs for health research. This indicated that people are content for their anonymous CDRs to be used in research, provided that appropriate safeguards are in place. However, study participants highlighted that the terms and conditions should be clearer, as should information to phone users on data collection, sharing, and uses in research [11].

Collating these ideas, our aim was to work toward an ethically founded framework for the socially acceptable use of mobile phone CDRs, particularly in health research. We propose that the design and findings of this study will have value in developing data governance guidelines for other areas of research using CDRs for public benefit and wider network applications. As such, in addition to MNOs and the research community, our target audience includes policy makers; funding bodies; ethical, scientific, and publication review committees; and others involved in shaping how person‑based data are used and protected.

### Approach

This study is based on a multifaceted approach that draws upon data governance arrangements in published health research using CDRs; public engagement and their information expectations from mobile operators; and data use scenarios of CDRs for health research, all against a backdrop of legislative and regulatory requirements.

We first briefly outline the legislative and regulatory backdrop to set the scene. The current European (EU) legislation for personal data and their usage is the General Data Protection Regulation (GDPR) 2016 (2016/679/EU) that came into force in May 2018 [12]. Other jurisdictions have similar legislation, and in addition, may have specific legislation such as the Privacy and Electronic Communications (EC Directive) Regulations 2003 (2002/58/EC) [13]. Essential to these frameworks is the requirement to use personal data within lawful provisions and to safeguard individual privacy. Importantly, the GDPR encompasses pseudonymized data in its scope of personal data. This is defined as follows:

Pseudonymisation means the processing of personal data in such a manner that the personal data can no longer be attributed to a specific data subject without the use of additional information, provided that such additional information is kept separately and is subject to technical and organisational measures to ensure that the personal data are not attributed to an identified or identifiable natural person.

13

This means that it is not necessarily sufficient to remove the commonly recognized identifiers from a dataset to render it anonymous, which will therefore have a bearing on the lawfulness of CDR data usage. Where data are not fully anonymous, general data processing can be carried out lawfully, provided that it can be justified under (at least) one of the provisions in Article 6 [12]. Crucially, although many jurisdictions have privacy legislation and regulations, they are often mutually inconsistent as a geographically bounded patchwork. This misalignment is problematic for networked operations, since they are necessarily international operations [14].

In addition to complying with all relevant legislation, data uses are found to abide by ethical principles with respect to individuals and the public. The Menlo report [15] set out four important principles for computer and information-security research, namely, beneficence, respect for persons, justice, and respect for the law and public interest. This report was important in shaping our study because it set out to show the new potential for risks to individual privacy with the expansion of information and communications technology-based research, unless there is a corresponding reinterpretation of ethical principles and their application, to provide the groundwork for ethically defensible research [15]. The four principles were explained by Letouzé [4] to demonstrate their relevance to CDR research:

1. Beneficence: understanding risks and benefits. Efforts should be made to maximize the probability and magnitude of research benefits while recognizing that the risks and benefits in using CDRs are not always immediately apparent and will depend on specific project objectives. Furthermore, commercial considerations need to be taken into account when framing the work.
2. Respect for persons. The issue of consent is gaining attention among privacy concerns on using and sharing CDR data. In general, there appears to be little provision to allow consumers to exclude unwanted uses of their data. Going forward, this issue should be considered, particularly with legislative changes due to the introduction of the GDPR.
3. Justice: bias and inequalities. This principle highlights the issues of equality and fairness in how risks and benefits are distributed. It is known that not everyone is able to contribute data and benefit from research using CDRs. Efforts should be made to understand and correct biases in CDR data to ensure that the findings are representative of society.
4. Respect for the law and public interest. There is a need to engage properly in legal due diligence. In addition, there is an ethical position to support social acceptability. This includes being transparent about how CDR data are collected, shared, and used and being accountable for actions taken.

The Organization for Economic Cooperation and Development guidelines set out eight principles for fair information practices. Briefly, these cover data collection limitations, data quality and relevance, purpose specification, data use limitations, security safeguards, openness, data subject rights, and accountability [16]. These principles, set out in 2012, are highly relevant to research using CDRs and other networked data, where as yet, there is no overarching ethical framework to safeguard individuals.

A review of the data governance arrangements in published studies was included in the design of our recent review [5]. The level of available detail varied, but we aimed to state whether data were anonymized or aggregated, the approval processes, and the data access arrangements. The purpose of this exercise was to assess current reported practice and learn from good examples. Although the majority of the studies we reviewed [5] used data from low- and middle-income countries, the researchers using the data were multinational, and MNOs operate internationally across jurisdictional boundaries. The public view on the use of CDRs for health research is an underresearched area, and our study is the only one known to date [11]. We draw upon the series of workshops we conducted with the public to incorporate their views into proposals for the ethical use of CDRs in research. There were four workshops; the first was a pilot (N=25) to inform the three subsequent groups (N=61 each). All the workshops were based in Wales, United Kingdom, and included a range of general public participants. Two were held at Swansea University and one at a further education college. The format included an initial questionnaire to gauge knowledge, a presentation of research using CDRs, a discussion of risks and benefits, and an exit questionnaire to assess postworkshop views.

In addition to relying on published research using CDRs, we created scenarios of CDR use with and without additional datasets in order to develop an indicative relative disclosure risk model. These were selected in discussion with local Health Board staff and based on questions of potential interest to the Board. There is a wealth of literature on anonymization and disclosure risk, drawing attention to the fact that just because data have been through a process of anonymization, they might not be immune to reidentification risks. We used the principles in the Anonymisation Decision‑Making Framework [17] to model the likely disclosure risks in our example scenarios. These principles take into account factors including dataset size, data accessibility (open vs controlled), data granularity, user knowledge, means available, and motivation. This guidance, discussions with Health Board staff, and our knowledge of working with data informed the development of the model. We then analyzed findings from a review [5], the public views [11], and the data use scenarios to propose a set of recommendations for the appropriate use of CDRs in health research.

## Results and Discussion

### Data Governance Arrangements in Published Studies

The extensive structured literature review on the use of mobile phone CDR data for health research revealed some common patterns in operational data governance regimes [5]. Datasets were provided to researchers at different levels of spatial granularity and over variously restricted time periods to mitigate risks. These varied by study or programs of study. In some cases, data were subjected to several layers of anonymization, and the true geolocations of cell phone masts were masked. The use of anonymized (or strongly pseudonymized) data was the norm, with few studies outside this model and with many additionally using aggregation and suppression of rare or extreme records. However, researchers reported that the use of overstringent measures sometimes precluded some potentially valuable research analyses. The requirement for formal ethical review varied, but proposals were routinely submitted to an internal ethics workgroup, and, in some cases, to an independent external group for wider considerations such as political implications, societal benefits, and risk versus utility. There were no known privacy breaches in relation to the data supplied for the studies included in the review [5].

The MNO Orange issued a Data for Development Challenge on mobile phone data based on 5 million Ivory Coast customers’ data over a 4-month period [18]. Researchers were tasked with using the data in a way that could potentially contribute to the socioeconomic development of the country. CDRs were anonymized by Orange Ivory Coast and processed by Orange Labs in Paris. In addition, the geographical locations of the cell phone masts were masked to protect the commercial interests of Orange. Four datasets of differing granularities were then released to researchers: (1) tower-to-tower data for the number and duration of calls between a pair of towers aggregated by each hour; (2) high spatial resolution data of individual movement trajectories on a random sample of 50,000 individuals for a 2-week time period only; (3) similar data for the entire observation period but with reduced spatial resolution to reduce the likelihood of identification; and (4) social network subgraphs using the CDRs generated by 5000 randomly selected individuals, divided into 2-week time periods [18]. A similar process was followed when Orange issued a second challenge, this time using data from Senegal. Orange established two ethics groups to review the proposals for data use: an internal ethics workgroup and an external panel of international experts [19,20].

For studies not involved with the Orange challenges, anonymized data were provided by the MNO apart from one study wherein researchers were provided with a raw dataset and undertook the anonymization process themselves [21]. High- and low-volume users were excluded from the analyses of this study to protect privacy. Another study stated that they followed the Groupe Spéciale Mobile Association privacy guidelines, which advise that any analyses on mobile phone records should be done using deidentified data and that individual-level data should not leave the MNO servers [22]. In this case, the data were analyzed remotely, and only aggregated data were released externally. Other studies explicitly stated that they used only aggregated data in their analyses. Where studies used additional datasets, they tended to integrate these with, or overlay them onto, the CDRs rather than use individual-level data linkage. In addition to ethical review by the MNO (where present), some studies reported seeking additional ethical approval from their institution before beginning their research study.

### Pubic Views and Concerns

A variety of concerns about CDR usage were expressed during the public workshops. Participants were worried about location data being sold to (potential) employers or insurers and the risk of disclosure for individuals living in remote areas. There were questions about whether data were being anonymized and aggregated to a sufficient standard and whether informed consent had been gained for identifiable data to be collected in the first place. Perceived risks included disclosure and data misuse and the increased possibility of reidentification from the use of multiple datasets. Participants made suggestions for what could be done to address these concerns; the most frequent was to have transparent terms and conditions, followed by provision of more information to phone users on the types of data being collected and how they are used. Stronger information governance featured highly, with a need for more legal sanctions for data misuse, the option to opt out, and the provision of more information on governance and security processes. The majority felt that information about the use of anonymized mobile phone data for research should be included in the terms and conditions [11].

### Relative Risk Model for Use of Call Detail Records

Data modeling exercises using semihypothetical scenarios were carried out to suggest a relative risk model for using CDRs in conjunction with publicly available or restricted access data. We based this on the information about CDR use from our review, as we did not have access to these data. The assessments took into account a number of assumptions. In terms of anticipated benefits, it is acknowledged that a phone might be carried by someone other than the contract holder and any MNO population coverage is partial; therefore, these issues may affect generalizability. Additionally, at least some of the research questions could be addressed by means other than using CDR data. However, the use of mobile phone data is an emerging area of work and presents novel opportunities, the full extent of which has not yet been realized. The outcome of the data modeling exercises is an indicative model, as it is not possible to assess absolute risk from this process. The risk estimates are based on the concept of reasonable effort, rather than what could be ascertained by a highly motivated, malicious intruder, but they do factor in potential repercussions due to the release of CDR data. We used six types of scenarios for illustration, some based on macro CDR data and some based on small-cell CDR data, but of course, other combinations are possible. Macro CDR location data are based on tower-to-tower communications and triangulation. Small cells are devices placed by MNOs within venues to capture local granularity, for example, inside a building or shopping mall. The scenarios were macro CDR data alone, small-cell CDR data alone, macro CDRs with publicly available datasets, small-cell CDRs with publicly available datasets, macro CDRs with restricted-access datasets, and small-cell CDR data with restricted-access datasets. It is assumed that all data are used in anonymized form, but in practice, MNOs often also aggregate the data for further risk mitigation. Publicly available datasets (such as weather, pollution levels, or disease incidence) are taken as aggregated. Restricted-access datasets (such as health records from hospitals or primary care) may be anonymized or aggregated depending on permissions and approvals. Details of the scenarios with their likely indicative risk profiles are shown in Multimedia Appendix 1. Considering these scenarios, we suggest the following relative disclosure risks:

1. Macro CDR data: It is considered highly unlikely that the majority of studies based solely on macro-level CDR data would present a significant risk to individual or group identity disclosure. There is a small possibility that the identity of some individuals in a dataset could be compromised through the use of location data patterns in CDRs, but this would require significant specialist effort. Provided that extreme records or very rare conditions/events are excluded, the risks of macro CDR–based studies should be low.
2. Small-cell CDR data: The sole use of small-cell CDR data in research studies is considered unlikely to present a significant risk to individual or group identity disclosure, unless particular groups, rare/sensitive conditions, or rare events are being studied. Because of the small geographies involved, the risks of working with small-cell CDR­–based data are likely to be low or low-moderate, depending on the nature of the study.
3. Macro CDR data with publicly available datasets: The use of macro CDR data in conjunction with publicly available datasets is considered unlikely to present significant risk to individual or group identity disclosure, unless particular groups, rare/sensitive conditions, or rare events are being studied via the association of more than one temporospatial dataset. The indicative risk level is therefore considered to be low-moderate.
4. Small-cell CDR data with publicly available datasets: It is considered that the use of small-cell CDR data in conjunction with publicly available datasets may present significant risk to individual or group identity disclosure. This is because, although the data are not linked at the individual level, it may be possible to associate the datasets temporospatially and thus to home in on small number (or individual) records due to the use of specific locations. The indicative risk level is therefore considered to be moderate.
5. Macro CDR data with restricted-access datasets: The use of macro CDR data in conjunction with restricted-access datasets is considered likely to present significant risk to individual or group identity disclosure. This is because of the sensitivity and granularity of the restricted-access data (such as general practice or hospital records), even if the data are not linked at the individual level. The model of access for the use of such combinations of data should be given serious consideration, with access within a data-safe haven likely to provide greater safeguards than external data release. The indicative risk level is therefore considered to be moderate to high.
6. Small-cell CDR data with restricted-access datasets: This scenario is similar to point number 5 mentioned above, but presents greater risks because of the use of small-cell CDR data in conjunction with restricted-access datasets. As such, it is considered highly likely to present significant risk to individual or group identity disclosure. This is because more specific locations are used along with individual health records, and consequently, access to such combinations of data should be given the utmost consideration. The indicative risk level is considered to be high.

The resulting indicative relative disclosure risk model is shown in Table 1.

### Recommendations for the Use of Call Detail Records for Public Benefit

Drawing the information together from the combined findings of the review [5], public views [11], and data use scenarios, we propose the following recommendations to promote the safe, socially acceptable use of mobile phone CDR data for public benefit. While recognizing the need to protect commercial interests, we propose that these interests be taken into consideration to contribute toward an ethically founded practical framework for developmental initiatives and research programs involving the use of CDR data wherever the work takes place. These recommendations are for MNOs and stakeholders—all the people involved in shaping how CDR data are used and protected:

1. The anticipated public benefit of the proposed data use should be clearly articulated and be sufficient to justify the potential risks of harm to data subjects.
2. All relevant legislative, regulatory, and governance approvals and data provider permissions should be sought with due diligence at the planning stage to ensure compliance.
3. Clear information (embodied in contract, as necessary) should be provided to stakeholders on expectations and responsibilities. This should include a description of the proposal, the placement of any small cells, and likely risks and benefits, so that informed decisions can be made by responsible parties.
4. Public views and preferences should be taken into account, beyond the strict requirements of legislation, to promote inclusivity and social license in the use of CDR data.
5. Learning from other initiatives and sharing good practice in data governance for the use of CDRs should be an ongoing process to avoid ethical pitfalls.
6. Due regard should be paid to modeling likely risks in research scenarios using CDR data with or without additional datasets on a case-by-case basis, so that appropriate safeguards at data access and release of results can be applied accordingly.
7. Transparency should be a fundamental consideration when communicating the likely risks and benefits of CDR data usage, including anticipated public benefit and lessons learned to inform future work.
8. Efforts should be made to engage with the public to provide information on the research uses of CDR data, potential public benefits, and possible opportunities to be involved in influencing the research agenda.
9. The terms and conditions of service should be made more accessible and clearer to data subjects in terms of design and content as part of a transparent data governance model.
10. The use of the best quality data at a granularity that enables the research question to be addressed without compromising privacy and security should be promoted.
11. Appropriate privacy notices on what data are collected; in which formats they are used; and for what purposes, by whom, and to whom they are sold/shared should be provided.
12. A high priority should be placed on having a robust, flexible data governance framework and being overtly accountable and responsible across all operations pertaining to the use of person-based data.

**Table 1:** Indicative relative disclosure risk model.

Data	Implication	Indicative level
Macro CDR^a^ data alone	Highly unlikely to present a significant risk to individual or group identity disclosure	Low
Small-cell CDR data alone	Unlikely to present a significant risk to individual or group identity disclosure	Low-moderate
Macro CDR data with publicly available datasets	Unlikely to present a significant risk to individual or group identity disclosure	Low-moderate
Small-cell CDR data with publicly available datasets	May present a significant risk to individual or group identity disclosure	Moderate
Macro CDR data with restricted datasets	Likely to present a significant risk to individual or group identity disclosure	Moderate-high
Small-cell CDR data with restricted datasets	Highly likely to present a significant risk to individual or group identity disclosure	High

^a^CDR: call detail record

### Summary Discussion

CDRs have been used successfully in a wide variety of health geography studies, with varying data governance regimes. Engagement with the public drew out valuable issues on the social acceptability of using CDRs in research. The relative risk model sets out a hierarchy of likely disclosure in different data use scenarios. Although this is only indicative, it can be used as a guide to inform data use proposals on a case-by-case basis. The resulting set of recommendations is a culmination of all aspects of the work to contribute to a framework for the safe, socially acceptable use of CDRs in research. Concerns have been raised regarding the ethics of using mobile phone data in research and the potential threat to privacy. This, for the most part, has stemmed from the absence of any clear ethical and regulatory framework to guide this type of research and the use of networked data more broadly [4,14]. The recommendations can be taken forward to address this identified lack of consistency. These recommendations are in accordance with the Menlo and Organization for Economic Cooperation and Development guidance [15,16] and consolidate their principles to add specific guidance in relation to the use of CDR data for research by adding the previously nonexistent evidence base gained via a review of data governance in published research, modeling risks in data use scenarios, and importantly, incorporating public views on the social acceptability of CDR data use.

Most published research reported results using anonymized data, and many went further to protect against reidentification by way of aggregation. Nevertheless, examples of how anonymization and aggregation do not guarantee privacy are abundant in the literature [23-27]. Moreover, breaches in group privacy do not rely on the reidentification of individuals. People who belong to certain groups on the basis of their gender, sexual orientation, ethnicity, or political preferences could become visible in CDR data and targeted [4,28]. Anonymization is a key concept in data protection legislation, and anonymized data are generally considered as data from which the data subject is no longer identifiable. However, the bar is set high in relation to the potential for reidentification and the risks related to this possibility. The essence of the EU GDPR is that if a data controller retains the key to reidentifying the data subject, the information should be more accurately considered pseudonymized rather than anonymized [29]. This has implications for the use of CDRs, as such data remain within the scope of the legislation. Depending on how the data are held and managed, it may be necessary for a data controller to justify that the data governance safeguards are in place and for the costs and effort involved in reidentification to be so remote or disproportionate that the data are effectively anonymized. This should include a consideration of physical, technical, and procedural controls around the data, for privacy by design, as well as controls applied directly to the data [17]. It is also important to consider the point at which data are anonymized and where this anonymization takes place. Data controllers have to comply with the GDPR if their work uses data pertaining to EU citizens, irrespective of whether their organization is based within the EU [13]. The GDPR also includes a requirement for transparent privacy notices on how data are collected and used, with relevant opt outs that are as easy to reverse as they are to agree [30]. As identified via our public workshops, clear, user-friendly, noncoercive information should be provided in the terms and conditions [11]. Furthermore, because of the finer granularity in data collection and possible risks, privacy notices should also be considered in locations where small cells are sited.

Proper ethical and societal principles are important for the use of person-based data, even when the data have been anonymized. Whichever data are to be used, due diligence should always be applied to ensure the proposed data uses are appropriate and safe. Generation of a digital footprint via active and passive data collection is increasing both in volume and scope. Mobile phones are among the most longstanding of networked devices, but many other data sources are coming to the fore via the IoT and other data-hungry advances [2]. It has been noted that when using our phones and other devices, we are trading our privacy for convenience, with many people finding the conventions of interaction obscure or inexplicable [31]. It has further been argued that with the increase in networked devices and the cultural and functional changes impacting society, as individuals, we will find ourselves in an “ethical bind” where we need to use the device, but cannot do so without surrendering data to the network [32]. Although it is appreciated that certain data are necessary for MNOs and other operators, there needs to be an ethical balance, so that individuals are not overlooked and treated as a commodity. For this reason, we need appropriate ethically founded data-governance frameworks with social acceptability in order to respect individual choice and use data for public benefit [17].

### What This Study Adds

This is the first-known study to use a multifaceted approach to propose recommendations for the ethically founded use of CDRs in research. Our findings are based on known practice in CDR use, public viewpoints, and a consideration of relative risk in data use scenarios. As such, it is a novel study that can be used as a model for other new and emerging personal data types in the increasingly networked digital world.

### Limitations

The main limitations of this study are related to the scenarios for data modelling, since we were unable to access the data in order to make a quantifiable risk assessment. This could be a topic for future work. However, it is worth pointing out that accurately quantifying disclosure risk from data use scenarios is not a trivial exercise, since it varies with many factors. As such, while recognizing that our model is only indicative, we recommend that data use proposals are assessed for risk on a case-by-case basis, making use of the principles proposed. In addition, although we chose to illustrate the model with six scenarios, other combinations are also possible. This could include a consideration of different health conditions, data volumes and granularities, access conditions, and other variables.

### Conclusions

This study has addressed the identified need for greater clarity and consistency in data governance for mobile phone CDRs in research. While recognizing that it is important to protect commercial interests, we propose that the recommendations be used to contribute toward an ethically founded practical framework to promote the safe socially acceptable use of CDR data for public benefit. With the increase in passive and active data collection from individuals using networked devices, this pattern needs to be repeated for the appropriate use of new and emerging data types from other applications and the wider IoT. Future work should consolidate these findings to assess the value of the recommendations as part of an ethically founded data-governance framework for the use of mobile phone CDR data and their wider applicability.

## Appendix

Multimedia Appendix 1 Scenarios of the use of call detail records for health research.

## Abbreviations

CDR: call detail record
EC: electronic communications
EU: European Union
GDPR: General Data Protection Regulation
IoT: internet of things
MNO: mobile network operator
