# Supplementary material for: Toward an Ethically Founded Framework for the Use of Mobile Phone Call Detail Records in Health Research
Source: JMIR Mhealth Uhealth. 2019 Mar 22;7(3):e11969. doi: 10.2196/11969 (PMC6450474; doi:10.2196/11969)
Supplement: Multimedia Appendix 1 [file mhealth_v7i3e11969_app1.pdf]

Multimedia Appendix 1. Scenarios in using Call Detail Records (CDRs) for health research.

No.	Scenario	Research question	Benefits	Data	Outputs	Considerations	Indicative relative risk
1	Using macro CDR data	Can macro CDRs be used to map where GP surgeries and other health clinics would be best placed?	It would be possible to map where high concentrations of people occur during usual clinic opening times to facilitate attendance	Macro spatio-temporal CDR data	Numbers of people filtered by gender and aggregated age group	Caution should be exercised if particular ages or ethnic groups are being studied	Low
2	Using small cell CDR data	How do the gender, age and interests of a mobile phone user vary during the day in people travelling past a potential advertising location?	This analysis could be used to target specific public health promotion messages to sub-populations	Small cell CDR data on people walking past a specific location per hour	Comparison of demographics, affluence profile, and interests	Care should be taken when targeting specific groups and/or rare conditions or phenomena	Low-Moderate
3	Using macro CDR data with publicly available datasets	Can macro CDRs be used to map the spread of measles reasonably accurately during an outbreak?	Understanding how disease spreads in relation to the mobility of people can inform public health strategies	Macro CDRs, along with public health measles outbreak data	Correlation between numbers and movement patterns over a given time period in relation to the temporo-spatial dispersion of measles	Associating two temporo-spatial datasets increases the relative risk of an individual being identified, particularly in rural areas where the number of cases might be low	Low-Moderate

4	Using small cell CDR data with publicly available datasets	Is there an increase of hospital attenders aged 60+ during the cold weather?	Informing health intervention development to target this sub-group to reduce hospital attendances	Small cell CDR data (via hospital-sited small cells) collecting gender, age and numbers of people per hour, used with weather reports	Correlation statistics on the association between people aged 60+ attending emergency departments with the weather	The sufficiency of safeguards should be considered when targeting specific groups and conditions in association with small-scale location datasets to ensure that disclosure risks are mitigated at data access and in the presentation of results	Moderate
5	Using macro CDR data with restricted access datasets	Is the number of asthma flare-ups and other respiratory problems associated with high air pollution periods and the numbers of people who have spent time in areas of high pollution?	Contributing to health service planning and resourcing, and informing public health campaigns	Macro level CDR data to show movement patterns over time, used with air quality monitoring data, hospital and primary care data	Correlation and goodness-of-fit statistics between the changes in levels of respiratory problems and concentrations of people in these areas	This scenario relies on being able to use restricted-access health data, in the form of hospital and GP records, along with macro-level CDRs and pollution data. It would depend on obtaining all relevant regulatory and governance approvals, and data provider permissions	Moderate - High
6	Using small cell CDR data with restricted access datasets	What are the reasons for attendance in A&E for people who have spent time in a night club district on a Saturday night?	This analysis would inform health board planning to reduce the burden on emergency departments during times when large	Small cell CDR data used with individual-level emergency department data	Summary of A&E attendances and their frequencies, with the	The considerations for no. 5 apply. But the relative risk to identity disclosure is significantly greater here, due to the granularity of location data plus individual-level health data	High

			volumes of alcohol may be consumed		reasons the visits occurred		
